# Differential Vpu-Mediated CD4 and Tetherin Downregulation Functions among Major HIV-1 Group M Subtypes

**DOI:** 10.1128/JVI.00293-20

**Published:** 2020-07-01

**Authors:** Gisele Umviligihozo, Kyle D. Cobarrubias, Sandali Chandrarathna, Steven W. Jin, Nicole Reddy, Helen Byakwaga, Conrad Muzoora, Mwebesa B. Bwana, Guinevere Q. Lee, Peter W. Hunt, Jeff N. Martin, Chanson J. Brumme, David R. Bangsberg, Etienne Karita, Susan Allen, Eric Hunter, Thumbi Ndung’u, Zabrina L. Brumme, Mark A. Brockman

**Affiliations:** a Faculty of Health Sciences, Simon Fraser University, Vancouver, British Columbia, Canada; b University of KwaZulu-Natal, Durban, South Africa; c Africa Health Research Institute, Durban, South Africa; d Mbarara University of Science and Technology, Mbarara, Uganda; e University of California, San Francisco, California, USA; f British Columbia Centre for Excellence in HIV/AIDS, Vancouver, British Columbia, Canada; g University of British Columbia, Vancouver, British Columbia, Canada; h Oregon Health and Science University-Portland State University School of Public Health, Portland, Oregon, USA; i Rwanda Zambia HIV Research Group-Projet San Francisco, Kigali, Rwanda; j Department of Pathology and Laboratory Medicine, Emory University, Atlanta, Georgia, USA; k Department of Global Health, Rollins School of Public Health, Emory University, Atlanta, Georgia, USA; l Emory Vaccine Center at Yerkes National Primate Research Center, Emory University, Atlanta, Georgia, USA; m Max Planck Institute for Infection Biology, Berlin, Germany; n Division of Infection and Immunity, University College London, London, United Kingdom; Ulm University Medical Center

**Keywords:** CD4, HIV-1, subtype, tetherin, Vpu, Downregulation

## Abstract

Downregulation of BST-2/tetherin and CD4 by HIV-1 viral protein U (Vpu) promotes viral egress and allows infected cells to evade host immunity. Little is known however about the natural variability in these Vpu functions among the genetically diverse viral subtypes that contribute to the HIV-1 pandemic. We collected Vpu isolates from 332 treatment-naive individuals living with chronic HIV-1 infection in Uganda, Rwanda, South Africa, and Canada. Together, these Vpu isolates represent four major HIV-1 group M subtypes (A [*n* = 63], B [*n* = 84], C [*n* = 94], and D [*n* = 59]) plus intersubtype recombinants and uncommon strains (*n* = 32). The ability of each Vpu clone to downregulate endogenous CD4 and tetherin was quantified using flow cytometry following transfection into an immortalized T-cell line and compared to that of a reference Vpu clone derived from HIV-1 subtype B NL4.3. Overall, the median CD4 downregulation function of natural Vpu isolates was similar to that of NL4.3 (1.01 [interquartile range {IQR}, 0.86 to 1.18]), while the median tetherin downregulation function was moderately lower than that of NL4.3 (0.90 [0.79 to 0.97]). Both Vpu functions varied significantly among HIV-1 subtypes (Kruskal-Wallis *P* < 0.0001). Specifically, subtype C clones exhibited the lowest CD4 and tetherin downregulation activities, while subtype D and B clones were most functional for both activities. We also identified Vpu polymorphisms associated with CD4 or tetherin downregulation function and validated six of these using site-directed mutagenesis. Our results highlight the marked extent to which Vpu function varies among global HIV-1 strains, raising the possibility that natural variation in this accessory protein may contribute to viral pathogenesis and/or spread.

**IMPORTANCE** The HIV-1 accessory protein Vpu enhances viral spread by downregulating CD4 and BST-2/tetherin on the surface of infected cells. Natural variability in these Vpu functions may contribute to HIV-1 pathogenesis, but this has not been investigated among the diverse viral subtypes that contribute to the HIV-1 pandemic. In this study, we found that Vpu function differs significantly among HIV-1 subtypes A, B, C, and D. On average, subtype C clones displayed the lowest ability to downregulate both CD4 and tetherin, while subtype B and D clones were more functional. We also identified Vpu polymorphisms that associate with functional differences among HIV-1 isolates and subtypes. Our study suggests that genetic diversity in Vpu may play an important role in the differential pathogenesis and/or spread of HIV-1.

## Abstract (precis)

The HIV-1 accessory protein Vpu enhances viral spread by downregulating CD4 and BST-2/tetherin on the surface of infected cells. Natural variability in these Vpu functions may contribute to HIV-1 pathogenesis, but this has not been investigated among the diverse viral subtypes that contribute to the HIV-1 pandemic. In this study, we found that Vpu function differs significantly among HIV-1 subtypes A, B, C, and D. On average, subtype C clones displayed the lowest ability to downregulate both CD4 and tetherin, while subtype B and D clones were more functional. We also identified Vpu polymorphisms that associate with functional differences among HIV-1 isolates and subtypes. Our study suggests that genetic diversity in Vpu may play an important role in the differential pathogenesis and/or spread of HIV-1.

## INTRODUCTION

The HIV-1 accessory protein Vpu is a multifunctional ∼16-kDa transmembrane protein that enhances viral infectivity and pathogenesis (1–4) by counteracting the antiviral effects of CD4 (5–7) and the host restriction protein tetherin, also known as BST2 or CD317 (8–10). Vpu-mediated downregulation of CD4 and tetherin on the infected cell surface facilitates the release of progeny virions and allows infected cells to evade aspects of the innate and adaptive immune responses. Removal of CD4 enhances virion infectivity by increasing Env incorporation into budding virions (11) and prevents CD4-induced changes in Env conformation that mediate antibody-dependent cell-mediated cytotoxicity (ADCC) (12–14). Antagonism of tetherin promotes virion release and dampens innate immune sensing processes triggered by budding virions that result in an NF-κB-dependent inflammatory response (15, 16). More recently, Vpu has been described to possess other activities that may enhance immune evasion and pathogenicity, including the following: downregulation of HLA-C (17), which allows infected cells to avoid recognition by cytotoxic T cells; downregulation of NK-, T-, and B-cell antigen (NTB-A) receptor (18), which protects infected cells from lysis by NK cells; and downregulation of T-cell immunoglobulin and mucin-domain containing-3 (Tim-3), which may enhance viral spread (19).

The highly diverse HIV-1 group M “pandemic” strains can be classified into 10 genetically distinct subtypes (A to D, F to H, J, K, and L) and nearly 100 circulating recombinant forms (CRFs) (20–23). It has been hypothesized that Vpu’s ability to counteract human tetherin was a key determinant in the ability of HIV group M strains to cross the species barrier from chimpanzees, since simian immunodeficiency virus (SIV) Nef proteins that antagonize their respective primate tetherin alleles do not counteract human tetherin (4). Indeed, of all primate lentiviruses capable of infecting humans, only the HIV-1 group M strains encode a Vpu protein that efficiently antagonizes human tetherin (4, 24). Functional differences attributable to naturally occurring viral sequence diversity have been demonstrated for a number of HIV-1 proteins (25–28), including viral accessory proteins (29–33). For example, a recent study of 851 Vpu sequences isolated from 14 individuals infected with HIV-1 subtype B revealed broad preservation of tetherin and CD4 downregulation functions despite extensive sequence variation (34). Other studies that assessed a limited number of natural Vpu isolates from HIV-1 subtypes A (35), B (36), and C (37) also reported maintenance of tetherin and CD4 downregulation activities, further supporting their central importance to Vpu’s role during infection. Globally, *vpu* ranks among HIV-1’s most diverse genes (38), but to our knowledge no studies have attempted to comprehensively assess variation in Vpu function using a large number of natural isolates representing diverse group M subtypes. A better understanding of HIV subtype-specific differences in Vpu function would improve our knowledge of crucial host/virus interactions and may identify new determinants of HIV-1 pathogenesis.

In this study, we examined the *in vitro* function of a diverse panel of 332 *vpu* isolates representing four major HIV-1 group M subtypes (A, B, C, and D), along with intersubtype recombinants or other uncommon strains, that were collected from chronically HIV-infected, antiretroviral-naive individuals. We observed marked subtype-specific differences in the ability of Vpu clones to downregulate CD4 and tetherin. We also identified Vpu polymorphisms that associate with functional variability among clones. Together, our results suggest that natural variation in Vpu may contribute to observed differences in HIV-1 pathogenesis or global spread.

## RESULTS

### Vpu sequence isolation and characterization.

We utilized existing plasma specimens from 332 individuals living with chronic HIV infection and naive to antiretroviral therapy, which were collected in Uganda (*n* = 151) (where subtypes A1 and D predominate), Rwanda (*n* = 24) (where subtype A1 predominates), South Africa (*n* = 71) (where subtype C predominates), and Canada (*n* = 86) (where subtype B predominates). Detailed methods for *vpu* isolation, cloning, and functional analysis were described previously by Rahimi et al. (39). Briefly, a single intact *vpu* sequence was isolated from plasma HIV RNA using universal primers optimized to amplify HIV group M subtypes. Each amplicon was cloned into pSELECT-RRE-GFP, which features independent promoters to drive expression of Vpu and green fluorescent protein (GFP), which was used as a transfection control. This plasmid was modified to encode the HIV-1 Rev-responsive element (RRE) motif downstream of the *vpu* cloning site (39), allowing native non-codon-optimized *vpu* sequences to be expressed following cotransfection with a plasmid encoding HIV-1 Rev.

**FIG 1:**
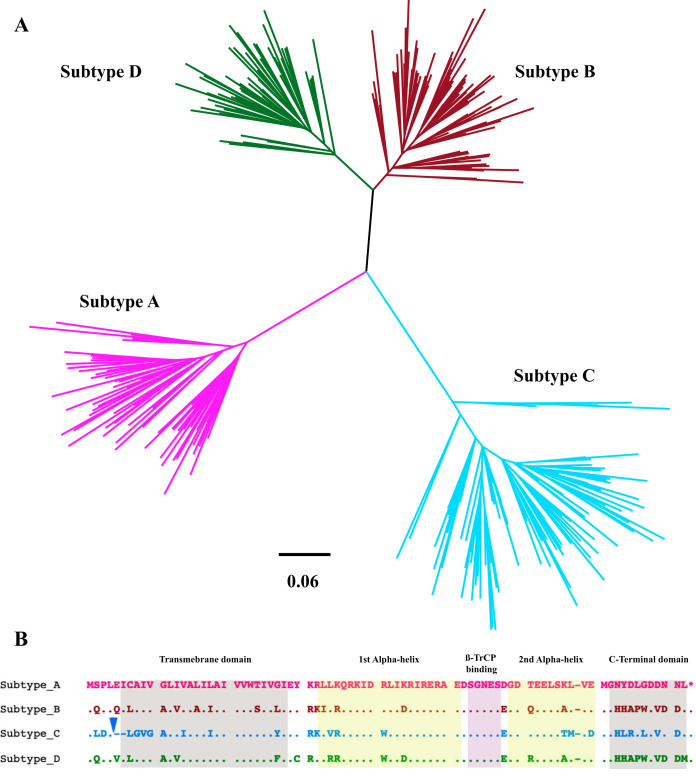
Vpu sequence diversity. (A) Maximum-likelihood phylogeny inferred from a nucleic acid sequence alignment of 300 HIV subtype A, B, C, and D *vpu* isolates analyzed in this study (32 *vpu* sequences encoding viral recombinants or other subtypes are not shown). Scale in estimated nucleotide substitutions per site. (B) Gap-stripped alignment of the Vpu consensus amino acid sequences for subtypes A, B, C, and D (defined as the most frequently observed residue at each position in our study sequences). Colors match the phylogeny in panel A. The inverted blue triangle denotes a common insertion that occurred exclusively in subtype C, usually seven amino acids in length (usually LA[K/R]VDYR). Major Vpu structural features are highlighted. β-TrCP, beta-transducin repeat-containing protein.

Genetic and phylogenetic analyses of *vpu* sequences confirmed that each isolate was unique and clustered with the original bulk plasma HIV RNA sequence for the participant, where available (data not shown). Of the 332 *vpu* sequences, 300 could be classified as belonging to subtype A1 (*n* = 63) (which is referred to as A in the remainder of this study), B (*n* = 84), C (*n* = 94), or D (*n* = 59) (Fig. 1A). The remaining 32 sequences comprised intersubtype recombinants (predominantly A/D or A/CRF01_AE), unclassifiable recombinants, and one subtype H sequence, which were grouped together as “other” Vpu clones for subsequent analysis. Intersubtype *vpu* diversity was substantial; aligned and gap-stripped subtype-specific amino acid consensus sequences are shown in Fig. 1B. Of note, subtype C *vpu* sequences uniquely harbored an insertion near the 5′ end, usually seven amino acids in length (most commonly LA[K/R]VDYR), followed by a two-amino-acid deletion (usually [E/Q/V]I, depending on the subtype comparison), making subtype C *vpu* sequences on average five amino acids longer than those of other group M subtypes. All subsequent analyses were based on Vpu amino acid sequences that were aligned and gap stripped as shown in Fig. 1B and also provided in Data Set S1 in the supplemental material.

Sociodemographic and clinical characteristics of the study participants are presented in Table 1, stratified by viral subtype. Median plasma viral loads were not significantly different between groups, but sex and age distributions differed markedly (all *P* < 0.0001). This is expected given the geographic diversity of the cohorts (e.g., the Canadian cohort was almost exclusively subtype B and consisted primarily of men who have sex with men, while the African cohorts featured all other subtypes and consisted primarily of females). Median CD4 cell counts also differed significantly between cohorts, with subtype D-infected individuals exhibiting the lowest values (median, 110 [interquartile range {IQR}, 54 to 188] cells/mm^3^) and subtype C-infected individuals exhibiting the highest values (median, 310 [IQR, 184 to 415] cells/mm^3^) (overall *P* < 0.0001). While we do not know the time of infection for participants, these observations are consistent with prior studies demonstrating that HIV subtype D displays more rapid disease progression in regions where it cocirculates with subtype A or C strains (40–43).

**TABLE 1:** Clinical characteristics of the study population

Parameter	Value for subtype:					*P* value
	A (*n* = 63)	B (*n* = 84)	C (*n* = 94)	D (*n* = 59)	Others (*n* = 32)	
Female sex, *n* (%)	35 (56)	6 (7)	64 (68)	41 (69)	19 (59)	<0.0001
Age in yr, median (IQR)	37 (32–40)	38 (33–45)	32 (27–37)	34 (30–38)	32 (25–39)	<0.0001
CD4 count in cells/mm^3^, median (IQR)	172 (103–249)	220 (130–340)	310 (184–415)	110 (54–188)	120 (64–112)	<0.0001
Log_10_ copies/ml plasma HIV-1 RNA, median (IQR)	4.97 (4.43–5.45)	5.0 (4.72–5.0)	4.8 (4.31–5.54)	5.11 (4.65–5.54)	4.8 4.48–5.12)	0.1173

### Variability in CD4 and tetherin downregulation function among diverse HIV-1 Vpu isolates.

**FIG 2:**
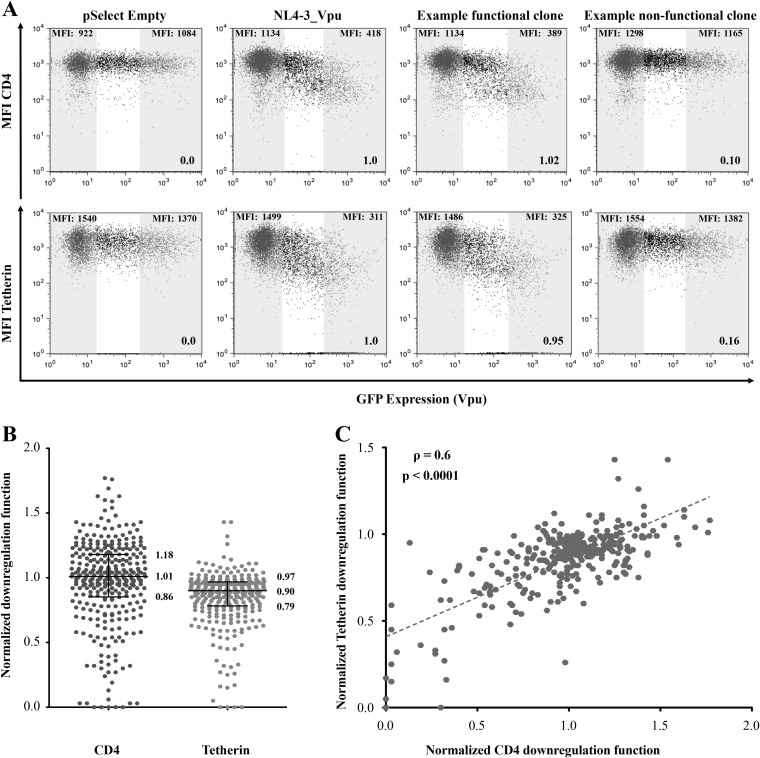
Analysis of CD4 and tetherin downregulation function. (A) Representative flow cytometry plots demonstrating downregulation of CD4 (top) or tetherin (bottom) following transfection of a negative control (pSelect empty vector), a positive control (NL4.3 Vpu), a representative functional clone, and a representative nonfunctional clone. Gray-shaded areas define GFP-negative (untransfected) and the GFP-high (Vpu-expressing) gates used for analysis. The median fluorescence intensity (MFI) of receptor expression is indicated at the top of each gate, and the downregulation function value (calculated as described in Materials and Methods) normalized to NL4.3 is indicated at the lower right. The absolute values of CD4 and tetherin reduction by NL4.3 Vpu were 69% ± 14% and 73% ± 13%. (B) Normalized CD4 and tetherin downregulation results for the 332 Vpu clones assessed in this study. Data are reported as the mean from at least three independent experiments. Horizontal lines and values report the median and interquartile ranges. (C) Association between CD4 and tetherin downregulation function for all 332 Vpu clones. Correlation was evaluated using Spearman’s rank sum test.

To examine *in vitro* Vpu function, each clone was transiently expressed in an immortalized CEM T-cell line, and its ability to downregulate endogenous CD4 and tetherin on the cell surface was assessed using flow cytometry. Representative data for one clone are shown in Fig. 2A. The median fluorescence intensities (MFI) of CD4 or tetherin in the GFP-negative (untransfected) cells versus the GFP-positive (Vpu-expressing) cells were quantified. All data were normalized to results for a negative control (empty vector) and a positive control (subtype B NL4.3 Vpu) analyzed in parallel, as described in Materials and Methods, such that Vpu function less than or greater than that of NL4.3 Vpu is indicated by values of <1 or >1, respectively. Both Vpu functions were assessed in a minimum of three independent experiments, and results are reported as the mean of all replicate measurements per clone.

The 332 Vpu clones exhibited a wide range of CD4 and tetherin downregulation functions, including some clones that were completely defective despite appearing to be genetically intact and others that exhibited more than 50% higher activity relative to NL4.3 Vpu (Fig. 2B). Despite this variability, median CD4 downregulation function of all Vpu clones was comparable to that of NL4.3 Vpu (1.01 [IQR, 0.86 to 1.18]). In contrast, median tetherin downregulation function of all Vpu clones was moderately lower than that of NL4.3 Vpu (0.90 [IQR, 0.79 to 0.97]). A relatively strong correlation was observed between CD4 and tetherin downregulation function among the diverse Vpu clones tested (Spearman ρ = 0.6; *P* < 0.0001) (Fig. 2C), suggesting that the mechanisms used by Vpu to modulate these cellular proteins may be partially overlapping. Alternatively, or in addition, intrinsic differences in protein expression, protein stability, or membrane localization may contribute in part to the observed variability in function. Although the majority of clones were functional for both downregulation activities, four clones were severely impaired (defined as the lowest 10th percentile) for both functions, while 15 clones displayed poor function (defined as the lowest 30th percentile) for one activity but normal function for the other. For example, clone CC255_UG_2007 was severely impaired for CD4 downregulation (relative function of 0.13), but its ability to downregulate tetherin was above average (0.95), while clone CC306_ZA_2012 was functional for CD4 downregulation (0.98) but impaired for tetherin downregulation (0.26). These observations are consistent with prior studies showing that Vpu uses distinct mechanisms to downregulate CD4 and tetherin (4) and further demonstrate that natural variation in Vpu can result in selective impairments in its function. The normalized CD4 and tetherin downregulation functions for each Vpu clone are provided in Data Set S1.

### Significant differences in Vpu function among HIV-1 group M subtypes.

**FIG 3:**
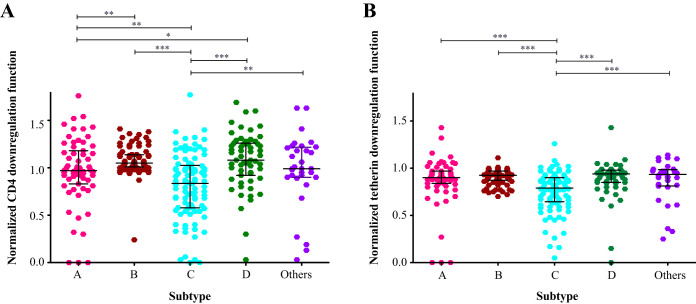
Differences in Vpu function among HIV group M subtypes. (A) CD4 downregulation activities of all Vpu clones, stratified by subtype. “Others” comprise non-A/B/C/D subtypes and unclassified/recombinant sequences. (B) Tetherin downregulation activities of all Vpu clones, stratified as in panel A. Horizontal lines denote median and interquartile ranges within. Significant variation was observed among groups for both data sets (Kruskal-Wallis *P* < 0.0001). Significant results based on pairwise Mann-Whitney U tests are indicated by asterisks: *, *P* < 0.05; **, *P* ≤ 0.01; ***, *P* ≤ 0.001.

Stratification of Vpu clones by viral subtype revealed significant differences in terms of their CD4 downregulation function (Kruskal-Wallis *P* < 0.0001) (Fig. 3A). Clones from subtype D demonstrated a greater ability to downregulate CD4 (median, 1.08 [IQR, 0.92 to 1.26]), followed by those from subtype B (median, 1.05 [IQR, 1.01 to 1.14]), other Vpu sequences including recombinants (median, 0.99 [IQR, 0.90 to 1.22]), and subtype A (median, 0.97 [IQR, 0.83 to 1.18]), while subtype C clones displayed the lowest function (median, 0.84 [IQR, 0.58 to 1.03]). In pairwise comparisons, the CD4 downregulation activity of subtype C clones was significantly lower than that of all other subtypes (*P* = 0.002 for subtype A, *P* = 0.005 for “other,” and *P* < 0.0001 for subtypes B and D). Furthermore, the downregulation function of subtype A clones was significantly lower than that of clones from subtype B (*P* = 0.003) or subtype D (*P* = 0.05).

Vpu clones from the different viral subtypes also varied significantly in their ability to downregulate tetherin (Kruskal-Wallis *P* < 0.0001) (Fig. 3B). Clones from subtype D (median, 0.94 [IQR, 0.85 to 0.98]) and “other” diverse clones (median, 0.94 [IQR, 0.81 to 0.99]) demonstrated the highest ability to downregulate tetherin, followed by those from subtype B (median, 0.93 [IQR, 0.87 to 0.97]) and subtype A (median, 0.90 [IQR 0.84 to 0.97]), while subtype C clones again displayed the lowest function (median, 0.79 [IQR, 0.65 to 0.90]). In pairwise comparisons, the tetherin downregulation function of subtype C clones was significantly lower than that of all other subtypes (all *P* < 0.001), while the function of clones from subtypes A, B, D, and “others” did not differ significantly from one another.

### Correlations between CD4 and tetherin downregulation function within subtypes.

**FIG 4:**
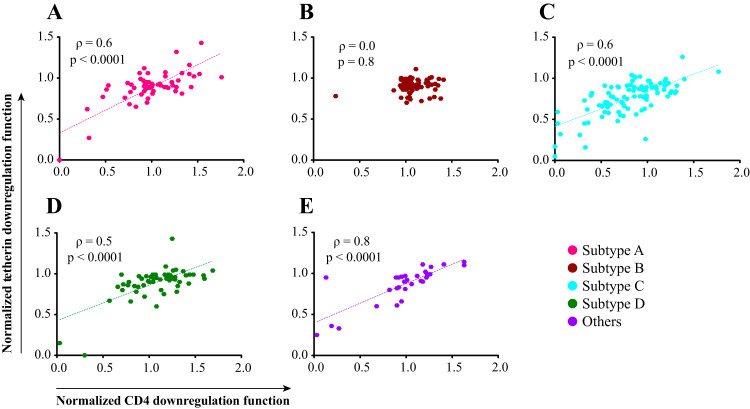
Association between Vpu downregulation functions. Correlation between the CD4 and tetherin downregulation abilities of each clone, stratified by subtype, is shown. Correlation was evaluated using the Spearman rank sum test.

Since the cellular mechanisms of Vpu-mediated CD4 and tetherin downregulation function are distinct (4), it is expected that genetic determinants of these Vpu functions would be to some extent separable. However, we observed a strong correlation between these two functions in our overall data set (Fig. 2C). To investigate this issue further, we stratified this analysis by subtype (Fig. 4). Overall, CD4 and tetherin downregulation activities correlated relatively strongly for Vpu clones from subtypes A, C, D, and “other” (all Spearman ρ ≥ 0.5; *P* < 0.0001). In contrast, no significant correlation was observed for subtype B (Spearman ρ = 0; *P* = 0.8), which differed from the other subtypes in that all clones except one displayed moderate to high function for both activities.

### Vpu polymorphisms associated with differential function.

**FIG 5:**
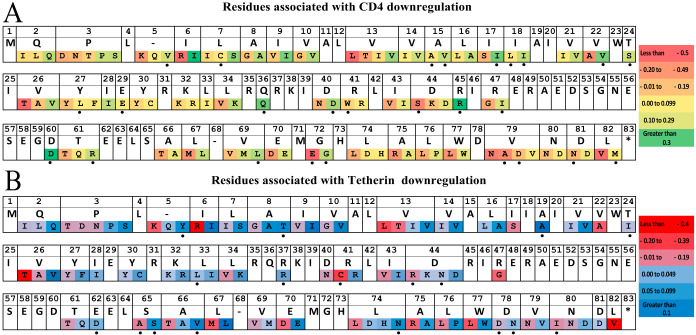
Vpu residues associated with variation in downregulation function. (A) Vpu residues associated with CD4 downregulation function. An aligned, gap-stripped Vpu consensus amino acid sequence, generated using all 332 *vpu* sequences in this study, is shown in the top row. Amino acids significantly associated with CD4 downregulation function (defined as at *P* < 0.05 and *q* < 0.1) are displayed in the bottom row in order of functional impact (from most negative to most positive). Dashes indicate gaps. Black dots denote residues uniquely associated with CD4, but not tetherin, downregulation at this statistical threshold. (B) Vpu residues associated with tetherin downregulation function, as described for panel A. Black dots denote residues uniquely associated with tetherin, but not CD4, downregulation at this statistical threshold.

**(i) Overall data set.** Given the rich sequence diversity represented in our data set, we undertook an exploratory sequence/function analysis to identify naturally occurring polymorphisms associated with Vpu downregulation activity using results from all clones (*n* = 332). Briefly, for every amino acid observed at least three times in the complete gap-stripped Vpu alignment, we employed the Mann-Whitney U test to compare the function of sequences harboring versus lacking the residue of interest. Multiple comparisons were addressed using a false-discovery rate (*q*-value) approach. At a predefined threshold of *P* < 0.05 and *q* < 0.1, we identified 120 amino acid substitutions, located at 52 distinct Vpu residues, that were associated with CD4 and/or tetherin downregulation function (Fig. 5). These included 103 substitutions, located at 47 distinct residues, that were associated with CD4 downregulation (see Data Set S2 in the supplemental material) and a highly overlapping set of 94 substitutions, located at 45 distinct residues, that were associated with tetherin downregulation (see Data Set S3 in the supplemental material). The substantial overlap is perhaps not surprising given the relatively strong correlations observed between CD4 and tetherin downregulation functions in our data set (Fig. 2C and 4). Of the 120 substitutions, 77 (64%) were concordant in their associations with both functions (42 were associated with lower CD4 and lower tetherin downregulation, while 35 were associated with higher CD4 and higher tetherin downregulation). The remaining 43 substitutions (36%) impacted one function only. No substitutions were identified that affected CD4 and tetherin downregulation in a discordant manner. These results are broadly consistent with those of a prior study of subtype B isolates (34), which also identified polymorphisms located at codons 43 and 67 that modulated both Vpu functions.

We were particularly interested to explore natural Vpu polymorphisms that are associated with larger functional impacts, since these may have outsized effects on viral pathogenesis. Overall, we identified 11 substitutions in our data set that were associated with a 30% or greater reduction in the median ability of Vpu to downregulate CD4 (Data Set S2). These were 26T (functional impact = −0.68), 13L (−0.57), 44S (−0.54), 6R (−0.53), 22A (−0.42), 5K (−0.36), 43V (−0.34) 13T (−0.32), 47G (−0.32), 72E (−0.31), and 34V (−0.3). Of these 11 polymorphisms, four (43V, 13T, 72E, and 34V) were relatively common (i.e., observed in 22, 7, 16, and 51 sequences in our data set, respectively), whereas the others were observed in five or fewer sequences. In contrast, only two substitutions were associated with a 30% or greater increase in the median ability of Vpu to downregulate CD4: 60D (+0.42; observed in 4 sequences) and 45R (+0.34; observed in 8 sequences).

Somewhat in contrast to CD4 downregulation, the per-substitution impacts of Vpu polymorphisms on tetherin downregulation function were less dramatic (Data Set S3). In total, nine substitutions were associated with a 20% or greater reduction in the median ability of Vpu to downregulate tetherin: 26T (functional impact = −0.45), 6R (−0.44); 41C (−0.32), 13L (−0.3), 22A (−0.26), 5K (−0.26), 47G (−0.25), 82V (−0.24), and 13T (−0.23). Of note, seven of these nine polymorphisms were also among those associated with the most dramatic reductions in CD4 downregulation function. No Vpu polymorphisms were identified as being associated with a 20% or greater increase in median tetherin downregulation function.

**(ii) Subtype-specific analysis.** Although our overall Vpu sequence/function analysis was relatively well powered at *n* = 332, the genetic diversity and function of Vpu clones varied markedly between HIV-1 subtypes. In particular, subtype C clones exhibited significantly poorer CD4 and tetherin downregulation activities and also encoded a number of distinctive residues compared to the other subtypes (Fig. 1B). Not surprisingly, many of the polymorphisms associated with poorer Vpu function could be identified as “signature” subtype C residues (e.g., 3D, 8G, 9V, 10G, 34V, 67M, and 70D, among others). To identify Vpu residues associated with function within each subtype, we repeated our sequence/function analyses for subtypes A, B, C, and D separately. For this, we used a more liberal statistical threshold of *P* < 0.05 and *q* < 0.2, recognizing that these data sets were substantially smaller (ranging from 59 to 94 sequences per group). Overall, 29 polymorphisms, located at 20 distinct Vpu residues, were identified as being associated with differential CD4 downregulation function in at least one HIV-1 subtype (see Data Set S4 in the supplemental material), where 14 (48%) of these polymorphisms were also identified in our overall analysis (Fig. 5). With the exception of 18L, which was associated with poorer CD4 downregulation function in both subtypes B and D (as well as in the overall analysis), little overlap was observed between subtypes. Furthermore, only two associations, 8T and 77W, were identified as being associated with tetherin downregulation function in the subtype-specific analysis, both of which were associated with higher downregulation activity in subtype B clones. Of these, 77W was also associated with higher tetherin downregulation function in the overall analysis (Fig. 5).

### Verification of impact of Vpu substitutions on CD4 and tetherin downregulation.

Our sequence/function analyses identified residues that were associated with differences in Vpu function, but these relationships are not necessarily causative. To confirm the impact of natural sequence variants on Vpu function, we focused on the polymorphisms that were associated with the most dramatic (≥30%) reductions in median CD4 downregulation function in our overall analysis (Data Set S2). Specifically, we selected 13L and 26T (which were associated with the greatest reductions in median Vpu function) plus 13T, 34V, 43V, and 72E (which reflected the four most frequently observed polymorphism with a functional impact of ≥ 30%). Five of these six residues (13L, 13T, 26T 34V, and 43V) were also associated with significant reductions in tetherin downregulation function in natural sequences (only 72E was not).

**FIG 6:**
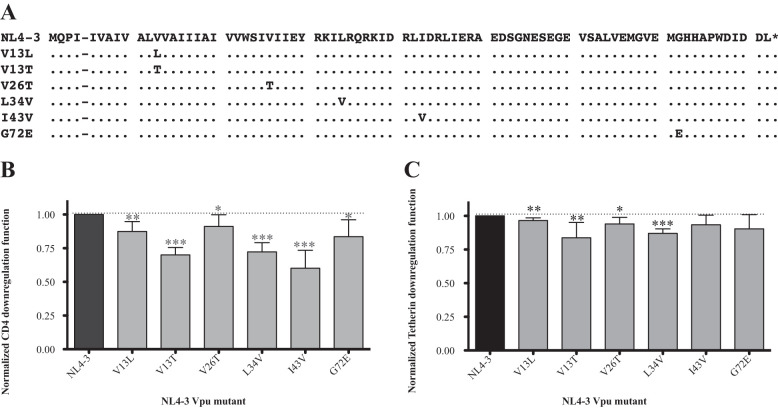
Experimental verification of residues associated with Vpu function. (A) Amino acid alignment for NL4.3 Vpu and six site-directed mutants. (B) Normalized CD4 downregulation function of each NL4.3 Vpu mutant. Bars denote mean and standard deviation, calculated from a minimum of 7 replicate measurements per mutant. Results were evaluated using the one-sample *t* test (with NL4.3 = 1.0), and significant differences are indicated by asterisks: *, *P* < 0.05; **, *P* ≤ 0.01; ***, *P* ≤ 0.001. (C) Normalized tetherin downregulation functions of each Vpu mutant, as described for panel B.

We introduced these six polymorphisms independently into the subtype B NL4.3 Vpu clone (Fig. 6A) and assessed each mutant for its ability to downregulate CD4 and tetherin in a minimum of seven replicate experiments. Consistent with our expectations, all six substitutions significantly reduced CD4 downregulation function: V13L by −0.13, V13T by −0.3, V26T by −0.10, L34V by −0.28, I43V by −0.4, and G72E by −0.16 (all *P* < 0.05) (Fig. 6B). Four out of the five substitutions that were predicted to reduce tetherin downregulation function also did so when engineered into NL4.3 Vpu: V13L by −0.03, V13T by −0.16, V26T by −0.07, and L34V by −0.13 (Fig. 6C) (all *P* < 0.05). Furthermore, the polymorphism that was expected to have no significant effect on tetherin downregulation function, G72E, displayed activity similar to that of the NL4.3 Vpu control. Of note, I43V, which was associated with reduced tetherin downregulation in natural sequences, did not significantly modulate the function of NL4.3 Vpu, indicating that its impact may be context dependent.

### Relationship between Vpu function and HIV clinical parameters.

**FIG 7:**
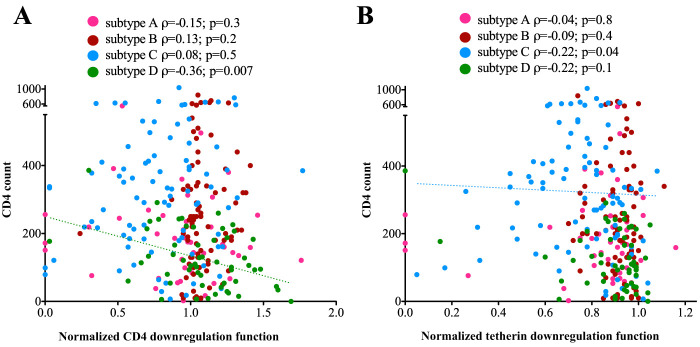
Relationships between Vpu function and CD4^+^ T-cell count, stratified by subtype. (A) Spearman’s correlations between normalized Vpu-mediated CD4 downregulation and CD4^+^ T-cell count, colored by HIV subtype. A dotted trendline is drawn to visualize the statistically significant relationship observed for subtype D. (B) Spearman’s correlation between normalized Vpu-mediated tetherin downregulation and CD4^+^ T-cell count, colored by HIV subtype. A dotted trendline is drawn to visualize the statistically significant relationship observed for subtype C.

Finally, given that the HIV-1 subtypes examined in our study are reported to display differential *in vivo* pathogenicity, we wanted to explore relationships between Vpu function and clinical parameters of HIV-1 infection, namely, CD4 count and plasma viral load (pVL). Given the substantial differences in clinical parameters and Vpu function between HIV-1 subtypes (Table 1 and Fig. 3), this analysis was performed in a subtype-specific manner. No association was observed between either Vpu downregulation function and pVL for any HIV-1 subtype (data not shown). However, we found a significant inverse correlation between Vpu-mediated CD4 downregulation function and CD4 count in subtype D (Spearman ρ = −0.36; *P* = 0.007) but not in the other subtypes (Fig. 7A). We also observed a weak inverse correlation between Vpu-mediated tetherin downregulation and CD4 count in subtype C (Spearman ρ = −0.2; *P* = 0.04) but not in the other subtypes (Fig. 7B).

## DISCUSSION

In this study, we examined the sequence and *in vitro* function of 332 Vpu isolates representing HIV-group M subtypes A, B, C, and D and recombinant strains. We demonstrate that natural sequence diversity in *vpu* is associated with marked variability in CD4 and tetherin downregulation activities, including among major group M subtypes (both *P* < 0.0001 by the Kruskal-Wallis test). While substantial intrasubtype variation was observed among Vpu isolates, a functional hierarchy emerged, with clones from subtypes D and B displaying better abilities on average to downregulate CD4 and tetherin, followed by those from subtypes A and C. Given the important role played by Vpu to enhance viral egress and evade host immunity, our results suggest that functional variation in this accessory protein might contribute to differences in pathogenesis that are observed among HIV group M subtypes (40–43). Indeed, the subtype C cohort on average exhibited the poorest Vpu-mediated CD4 and tetherin downregulation abilities (Fig. 3) but the overall highest CD4^+^ T-cell cell counts (Table 1). In an analysis stratified by HIV-1 subtype, we also found associations between Vpu function and CD4 cell count in subtypes C and D. While these associations were modest and did not point to a single Vpu mechanism, our study was neither designed nor sufficiently powered to address this question. Notably, we and others have observed that several HIV proteins derived from subtype C viruses display impaired *in vitro* function, including Gag, Pol, and Nef (29, 44, 45) suggesting that a less pathogenic disease course may contribute to the increased transmissibility seen for this subtype, which now accounts for approximately 50% of global HIV cases (22).

We used the linked data set derived from these 332 Vpu clones to explore potential sequence determinants of Vpu function. In total, we identified 120 amino acid polymorphisms, located at 52 distinct Vpu residues that were associated with CD4 and/or tetherin downregulation function. While we confirmed six of these polymorphisms through site-directed mutagenesis of NL4.3 Vpu, more research is needed to validate these observations. This correlative analysis should be considered exploratory, since the impact of individual polymorphisms is likely to be highly context dependent, but it may nevertheless provide some biological insights. Prior work by Pickering et al. (34) that examined the *in vitro* function of 304 subtype B Vpu isolates collected from 14 individuals identified mutations at three residues (I17T, V22A, and I39L) in clones displaying a selective impairment in CD4 downregulation and at 10 residues (I9M, A15V/T, I16E, A19E, E48K/G, N55H, E56G, E63A, S65A, and W76) in clones displaying selective impairment in tetherin counteraction (based on virion release assays. While similar in terms of the number of Vpu clones assessed, our study incorporated isolates from more individuals (*n* = 332) and included several non-B subtypes, which substantially increased the sequence and functional diversity of the clones examined. Consistent with the study by Pickering et al., we found that many Vpu polymorphisms were associated with a reduction in both CD4 and tetherin downregulation function. This suggests that these polymorphisms affect aspects of protein stability or intracellular protein localization that may have nonspecific impacts on Vpu function or, alternatively, that they are located in a domain that is of shared importance for both Vpu functions. In total, we identified 26 Vpu polymorphisms, located at 23 residues, that were associated with modulation of CD4 downregulation function only; for example, sequences containing I17 and V22 in the transmembrane domain exhibited higher function (+0.19 and +0.18, respectively) than sequences with other residues at these sites. In addition, we identified 17 polymorphisms, located at 15 residues, that were associated with modulation of tetherin downregulation only; for example, sequences containing A19 in the transmembrane domain and S65 in the second α-helix domain exhibited higher function (+0.11 and +0.07, respectively) than sequences with other residues at these sites. Notably, while the impact of S65A on Vpu function was modest (−0.07), this polymorphism was observed in 75 (∼23%) Vpu isolates, making it one of the more prevalent variants in our study. Our mutagenesis analysis focused on validating polymorphisms that were expected to have greater impacts on Vpu function, but future studies should also consider the relatively larger number of more prevalent polymorphisms that may have moderate functional impacts, since these might show additive effects in circulating isolates. It is also important to recognize the potential impact of genetic overlap between the *vpu* and *gp120* reading frames: the latter's ATG start codon lies between *vpu* codons 55 (N) and 56 (E). We observed a relatively large number of functional associations in this region of overlap (amino acids 55 to 83), suggesting that adaptive changes in one of these proteins could impact the other.

Several limitations of this study should be mentioned. First, we have only examined the ability of Vpu clones to downregulate CD4 and tetherin from the cell surface, which may be insufficient to make broad conclusions on Vpu’s potential clinical impact. While a subset of clones described here were assessed previously for their ability to downregulate HLA-C (46), we have not comprehensively assessed this function for the entire panel. We have also not explored other cellular proteins that are targeted by Vpu, such as NTB-A (18) or Tim-3 (19), and we have not examined Vpu’s ability to counteract tetherin-mediated NF-κB signaling, which may require protein motifs that are distinct from those involved in tetherin downregulation. Additional research will be needed to address these important topics; however, this work can be facilitated by our panel of 332 diverse Vpu clones. Second, to maximize our coverage of global Vpu diversity, we isolated a single Vpu clone per participant; as such, our study does not address within-host Vpu functional diversity. Finally, we employed an *in vitro* cotransfection-based assay to express native Vpu sequences linked to the RRE motif in the presence of HIV Rev and quantified endogenous CD4 and tetherin on the cell surface using flow cytometry. While this assay is robust and moderately high throughput, it may not fully recapitulate Vpu expression or function during viral infection, where it acts in concert with other viral proteins to modulate CD4. Nevertheless, our results advance our knowledge of Vpu sequence and functional diversity, particularly for Vpu clones derived from non-B subtypes.

In summary, we have demonstrated that Vpu-mediated CD4 and tetherin downregulation function differs among HIV group M subtypes A, B, C, and D. Our results highlight the potential importance of natural variation in Vpu in HIV pathogenesis and/or spread.

## MATERIALS AND METHODS

### Study specimens and approvals.

In total, 332 archived plasma specimens from HIV-infected antiretroviral-naive participants were obtained from cohorts located in Vancouver, Canada (HAART Observational Medical Evaluation and Research [HOMER] study), Mbarara, Uganda (Uganda AIDS Rural Treatment outcomes [UARTO] study), Kigali, Rwanda (Rwandan Heterosexual Transmission [HT] study), and Durban, South Africa (Sinikithemba cohort). All participants provided written informed consent. This study was approved by the research ethics boards (REBs) at Simon Fraser University (Canada) and the University of British Columbia/Providence Health Care (Canada).

### Vpu amplification, sequencing, and subtype determination.

HIV-1 RNA was extracted from plasma using the NucliSENS EasyMag system (bioMérieux), and the region containing *vpu* was amplified using the Superscript IIITM one-step reverse transcription-PCR (RT-PCR) system with Platinum *Taq* HiFi (Invitrogen). For specimens from Canada, Rwanda, and Uganda, degenerate primers were designed to capture HIV-1 group M *vpu* sequence diversity, as follows: forward, 5′-TTGGGTGYCR RCAYAGCAGR ATAGG-3′ (representing nucleotides 5780 to 5804 of the HIV-1 subtype B genomic reference strain HXB2); reverse, 5′-ATRTGCTTTV GCATCTGATG CACARAATA-3′ (representing HXB2 nucleotides 6407 to 6379). For specimens from South Africa, degenerate primers were designed to capture HIV-1 subtype C *vpu* sequence diversity, as follows: forward, 5′-CTTAAGACAG CAGTACAAAT GGCAGT-3′ (representing HXB2 nucleotides 4743 to 4768); reverse, 5′-GCATCTGATC CACCATGTCA TTTTYCC-3′ (nucleotides 6537 to 6511 of HXB2). RT-PCR amplicons were subjected to nested PCR using the Expand High-Fidelity Plus PCR system (Roche) employing primers that were optimized to capture HIV-1 group M diversity that featured restriction sites for cloning, as described by Rahimi et al. (39). For this, our primary forward primer (5′-AGAGGGCGCG CC**ATCAARHT YCTVTAYCAA AGCAGTAAGT A**-3′; the AscI site is underlined, and bold bases span HXB2 nucleotides 6024 to 6052) was located 38 bases upstream of the *vpu* start site. The reverse primer was 5′-GCCTCCGCGG ATCGAT**GGTA CCCCATARTA GACHGTRACC CA**-3′ (SacII and ClaI sites are underlined; bold bases span HXB2 nucleotides 6352 to 6327). Amplicons were Sanger sequenced on a 3130xl or 3730xl automated Genetic Analyzer (Applied Biosystems Inc.), and chromatograms were analyzed using Sequencher v5.0.1 software (GeneCodes). Intact clonal and original bulk *vpu* sequences were aligned using HIV Align (47) hosted at the Los Alamos National Laboratory (LANL) HIV sequence database (www.hiv.lanl.gov/content/sequence/VIRALIGN/viralign.html) and manually edited using Aliview (48). Maximum-likelihood phylogenies were inferred from *vpu* sequence alignments using PhyML (49) and visualized using FigTree v1.4.3 (50). HIV-1 subtype determination was performed using the Recombinant Identification Program (51) at the LANL database (www.hiv.lanl.gov/content/sequence/RIP/RIP.html) using a window size of 100 and a confidence threshold of 95%, as well as by visual inspection of phylogenies.

### Vpu cloning.

Our method to clone and express natural Vpu sequences has been described previously (39). Briefly, the eukaryotic expression vector pSELECT-GFP (InvivoGen), which features dual promoters that independently drive expression of GFP and the gene of interest, was modified to encode the HIV-1 Rev response element (RRE) downstream of the Vpu cloning site, generating pSELECT-RRE-GFP (39). Second-round *vpu* amplicons were column purified using the E.Z.N.A Cycle Pure kit (Omega Bio-tek) and ligated into pSELECT-RRE-GFP using AscI and SacII sites (New England Biolabs). The ligation products were transformed into E. cloni 10G chemically competent cells (Lucigen), plated on LB agar plates containing Zeocin, and then grown at 37°C overnight. For each specimen, a single colony was isolated and grown in LB medium containing Zeocin at 37°C for ∼18 h. The plasmid DNA was purified using the E.Z.N.A plasmid minikit (Omega Bio-tek) and Sanger sequenced as described above.

### *In vitro* analysis of CD4 and tetherin downregulation by flow cytometry.

To examine Vpu downregulation function, 1.5 μg of pSELECT-Vpu-RRE-GFP and 2 μg of pSELECT-Rev were cotransfected into 500,000 CD4^+^ CEM T cells by electroporation in a total volume of 50 μl Opti-MEM I medium (Life Technologies) using a Bio-Rad GenePulser MXCell instrument (96-well plate; single 25-ms square-wave pulse at 250 V, 2,000 μF, infinite Ω). Transfected cells were resuspended in 350 μl R10+ medium (RPMI 1640 supplemented with 2 mM l-glutamine, 1,000 U/ml penicillin, and 1 mg/ml streptomycin, all from Sigma-Aldrich) plus 10% fetal bovine serum (Life Technologies) and incubated for 20 h at 37°C with 5% CO_2_. After incubation, 250,000 cells were stained with antibodies allophycocyanin (APC) anti-human CD4 (clone A161A1; BioLegend) and phycoerythrin (PE) anti-human CD317/BST2/tetherin (clone RS38E; BioLegend) at 4°C for 30 min, washed twice, and resuspended in 250 μl phosphate-buffered saline (PBS) solution (Sigma-Aldrich). Cells were analyzed on a Guava EasyCyte 8HT flow cytometer (Millipore) and quantified using FlowJo 9.9.6 software. Sample gating was standardized using positive (NL4.3 Vpu) and negative (empty pSELECT-RRE-GFP) controls. The ability of each clone to downregulate CD4 or tetherin was expressed by the median fluorescence intensity (MFI) of CD4 or tetherin in the GFP-high (i.e., Vpu-expressing cells) versus GFP-negative (i.e., untransfected cells) gates. This value was then normalized to that of the positive control (NL4.3 Vpu) examined in parallel using the formula [1 − (MFIclone/MFINeg)]/[1 − (MFINL4.3/MFINeg)], where MFIclone refers to the surface expression of CD4 or tetherin in cells transfected with a Vpu clone of interest, MFINeg refers to surface expression in untransfected cells, and MFINL4.3 refers to surface expression in cells transfected with the NL4.3 control. As such, values of <1.0 or >1.0 indicate functions that are less than or greater than that for NL4.3 Vpu, respectively. Each Vpu clone was assessed in a minimum of three independent experiments, and results are reported as the mean.

### Mutagenesis.

Overlap extension PCR was used to introduce mutations into the HIV-1 subtype B NL4.3 Vpu reference strain, as described previously (52). Amplicons were gel purified using GeneJET (Thermo Fisher Scientific) and confirmed by Sanger sequencing as described above. Sequence-verified amplicons were cloned into pSELECT-GFP-RRE and assessed for their CD4 and tetherin downregulation functions as described above. Each mutant Vpu clone was tested in a minimum of seven independent experiments.

### Statistical analysis.

The Kruskal-Wallis test was used to compare Vpu functions between viral subtypes. Spearman's correlation was used to characterize relationships between Vpu functions, as well as between Vpu functions and HIV clinical parameters (pVL and CD4 count). Frequency data were analyzed using Fisher’s exact or Chi-square tests. The one-sample *t* test was used to compare the function of NL4.3 mutants to that of the parental strain (whose function was set at 1.0). Statistical analyses were performed using Prism v8.0 (GraphPad). A custom python script was used to apply the Mann-Whitney U test to assess relationships between every amino acid observed a minimum of three times at each position in the gap-stripped Vpu alignment and each of Vpu’s two functions. Here, multiple comparisons were addressed using *q* values (the *P* value analogue of the false-discovery rate), which is defined as the expected proportion of false positives among results deemed significant at a given *P* value threshold (e.g., at a *q* value of ≤0.2, we expect 20% of identified associations to be false positives) (53).

### Data availability.

All *vpu* sequences are available under GenBank accession numbers MT116441 to MT116772. The aligned, gap-stripped Vpu amino acid sequences, subtype classifications, and functional characteristics of clones are provided in Data Set S1 in the supplemental material. Vpu polymorphisms associated with function are provided in Data Sets S2, S3, and S4 in the supplemental material.

## Supplementary Material

Supplemental file 1

Supplemental file 2

Supplemental file 3

Supplemental file 4
